# Notes and News

**Published:** 1904-01-09

**Authors:** 


### Notes and News.

New wings have been added to the Royal Victoria Hospital and Hotel Dieu in Montreal recently.

The First International Congress on School Hygiene will be held at Nuremberg from April 4th to the 9th.

The London Homœopathic Hospital staff dinner will be held on January 14th at the Wharncliffe Rooms, Hotel Great Central.

Dr. Finsen has presented £2,753, part of the Nobel Prize awarded to him, to found a prize in connection with his Light Institute at Copenhagen.

The Buda Pesth Hospital has led the way in Hungary to the employment of female practitioners in hospitals by appointing Dr. Ida Szentesz as assistant physician.

On Thursday, 14th inst., at 20 Hanover Square, W., at 8 p.m., a paper will be read by Dr. Mendes de Leon before the British Gynæcological Society on "A hitherto not sufficiently recognised source of infection during operations." The President will deliver his valedictory address.

Some years ago such grave suspicion was cast upon the properties of watercress for conveying typhoid that amongst the well-to-do watercress has almost entirely fallen into disuse. But amongst the poorer classes the inexpensive relish has remained a favourite. Now the subject has been raised again owing to recent inquiries which revealed the fact that practically the whole of the watercress supply in Hackney is cultivated under polluted conditions such as may give rise to an epidemic of typhoid at any time. It would be well if the medical officer in every district could ascertain the source of supply with a view to securing pure watercress for the poorer population.

King's College has already received £100,000 towards the £300,000 required for its removal.

The presence of radium has been discovered in the waters of Bath, but in very minute quantities.

Two new wards for children were opened at the Mater Infirmorum Hospital, Belfast, on Christmas Day.

The Government Food Test Commission sitting at Washington has decided that the presence of salicylic acid in food is injurious to health.

Mrs. Jane Gabriel has presented £1,000 to Charing Cross Hospital to endow a bed in memory of her husband, the late Mr. Arnold Gabriel.

A well-known medical school and the hospital attached, in Japan, have been closed, because it is said that the educational department refused to allow it to be entitled a college which the proprietors wished to do.

Eighteen regimental homes have been completed and occupied by partially and entirely disabled soldiers invalided in South Africa. Many private individuals are assisting the scheme by presenting sites or houses in connection with certain regiments in which they take an interest.

The question of fruit culture in England which will be largely canvassed now that the Board of Agriculture are instituting an inquiry into the subject, is an important one to the nation. But the difficulties with which fruit-growers have to contend with in this country are so great that unless an effective organised system of collecting and distributing is introduced the foreign fruit will hold its own on account of the lower cost. Even perishable fruit like strawberries directly they become plentiful do not pay the growers owing to the labour—which, being casual, is difficult to procure and costly, and to the heavy charges for carriage on the railways.

The resignation of the Chair of Medicine at Oxford by Sir J. Burdon-Sanderson is a great loss to Oxford, if only regarded from the fact that the name of Burdon-Sanderson added to the prestige of the Medical School. The position of Regius Professor of Medicine at Oxford has been associated with such notable members of the medical profession that any attempt to render it less distinctive by amalgamation with an inferior post would be a grave mistake. There is a movement on foot to unite the post of Reader in Pathology with that of Professor of Medicine. This movement is being strenuously opposed by those who desire to uphold the dignity of the Oxford Medical School. It is felt that the Regius Professor of Medicine should possess a practical knowledge of the most modern requirements of clinical teaching and a recognised position and reputation of the highest order in his profession, and that his position should be distinct from one requiring teaching in detail.

